# 

**Published:** 1880-11

**Authors:** 


### CONTENTS.

### COMMUNICATIONS.

"Dental Ethics—Fee Bills—Tooth Powders and Mouth Washes—their Uses and Abuses," ... 471
The History of the Philadelphia Fraudulent Medical Colleges ... 476
The Restrospective View of Dentistry ... 481
Extracts from an Address ... 484
Cure for the Tobacco Habit ... 491
Synopsis of an Introductory Lecture to a Course of Dental Mechanics 492

### SELECTIONS.

The Population of the World ... 498
Dental Education in Hungary ... 499
Fur on the Tongue ... 501
Observations on the Secretion of Saliva in a case of Parotid Fistula ... 503
Method of Preserving the Brain ... 504
How our School makes Doctors ... 506

### EDITORIAL.

A Suggestion: ... 508
The Teeth of the Ancient Greeks ... 509
The Ohio State Dental Society ... 511
State Meeting ... 511
Examining Board ... 512
Revival ... 513
Bibliographical ... 513

